# Editorial: New Trends and Approaches in Perioperative Pharmacotherapy: An Update

**DOI:** 10.3389/fphar.2021.721075

**Published:** 2021-08-06

**Authors:** S. Soghomonyan, N. Stoicea, S. D. Bergese

**Affiliations:** ^1^ Department of Anesthesiology, Ohio State University Wexner Medical Center, Columbus, OH, United States; ^2^ Ohio State University College of Pharmacy, Columbus, Summa Health System, Akron, OH, United States; ^3^ Department of Anesthesiology, Renaissance School of Medicine, Stony Brook University, Stony Brook, NY, United States

**Keywords:** perioperative pharmacotherapy, postoperative recovery, anesthesia, adverse drug effects, enhanced recovery after anaesthetic, postoperative nausea and vomiting

The perioperative period poses significant additional risks on patients, who already suffer from various health-related problems. Even with advances in surgical treatment, anesthesia safety, diagnostic imaging, and intensive care, the perioperative period is still associated with serious morbidity and mortality. According to Bhatia et al. (2021), the 30-day mortality in patients undergoing non-cardiac surgery remains as high as 1.3–1.9%, and the incidence of postoperative myocardial infarction reaches 4.2–6.3%. Cerebrovascular and pulmonary complications, impaired glycemic control, metabolic derangement, infections, and iatrogenic complications all add up to the long list of potential risks and adverse effects encountered in the perioperative period (Ben-Shlomo and Melmed, 2003). Current approaches to perioperative patient care highlight the requirement for adequate pain control using multimodal therapy: opioids, non-opioid analgesics, gabapentinoids, pain modifiers, regional blocks, and other therapies. Post-surgical enhanced recovery protocols have been developed in recent years allowing for shortened recovery time and improved patient satisfaction.

Patients undergoing surgery commonly receive chronic treatment with anti-hypertensives, anti-arrhythmic drugs, anticoagulants, chemotherapeutic drugs, and other medications. Many of these drugs have the potential to cause serious drug-drug interactions and perioperative adverse effects (Pai et al., 2017; Pfeifer et al., 2021).

Undoubtedly, questions related with the perioperative patient care and drug management in this patient group are among the most discussed in medical literature.

The Society for Perioperative Assessment and Quality Improvement highlights the importance of evidence-based approach to perioperative care and medication management (Pfeifer et al., 2021).

Taking into account all these trends and advances in patient care, as well as advances in surgical care and complexity of surgical procedures, our editorial team decided to invite medical professionals to share with the readers their clinical experience and the results of their research in the constantly changing field of perioperative pharmacotherapy. Our decision was also based on success of our previously published topic dedicated to perioperative pharmacotherapy, which was viewed over 109,000 times.

The current update of our topic is an attempt to put together the recent advances in the field of perioperative pharmacotherapy and present opinions and results of health care professionals from different hospitals and countries.

The manuscripts that were included in the topic cover several important aspects of patient care. Questions discussed include drug interactions and adverse drug reactions during anesthesia, coagulation management in patients undergoing liver transplant surgery, interaction of cigarette smoking and drugs used during anesthesia, efficacy of antifibrinolytic therapy in major spinal surgery, use of vasopressors in free tissue transfer in head and neck surgery, and many other important problems.

The efficacy of prevention and treatment of postoperative nausea and vomiting (PONV) is an important component of perioperative care and one of the criteria to assess quality of care. It is well known that many drugs used in PONV management prolong the QTc on the EKG. However, there are insufficient data in literature related to PONV management in patients who present with an already prolonged QTc. A mini-review in our topic presents this important question attempting to focus the attention of clinicians and researchers on this important problem and encouraging further discussion and research to find an optimal strategy for PONV management in this patient category.

As a conclusion, surgery and anesthesiology are actively changing disciplines, and effective treatment of patients undergoing surgery is only possible with an up-to-date knowledge of the ongoing trends and advances in perioperative pharmacotherapy.
